# Defining Pathological Activities of ALK in Neuroblastoma, a Neural Crest-Derived Cancer

**DOI:** 10.3390/ijms222111718

**Published:** 2021-10-29

**Authors:** Anna M. Wulf, Marcela M. Moreno, Chloé Paka, Alexandra Rampasekova, Karen J. Liu

**Affiliations:** 1 Centre for Craniofacial and Regenerative Biology, King’s College London, London SE1 9RT, UK; anna.wulf@kcl.ac.uk (A.M.W.); marcela.marques_moreno@kcl.ac.uk (M.M.M.); chloe.paka@kcl.ac.uk (C.P.); alexandra.rampasekova@kcl.ac.uk (A.R.); 2 STEMCELL Technologies, Cambridge CB25 9TL, UK

**Keywords:** neuroblastoma, anaplastic lymphoma kinase, ALK, neural crest

## Abstract

Neuroblastoma is a common extracranial solid tumour of childhood, responsible for 15% of cancer-related deaths in children. Prognoses vary from spontaneous remission to aggressive disease with extensive metastases, where treatment is challenging. Tumours are thought to arise from sympathoadrenal progenitor cells, which derive from an embryonic cell population called neural crest cells that give rise to diverse cell types, such as facial bone and cartilage, pigmented cells, and neurons. Tumours are found associated with mature derivatives of neural crest, such as the adrenal medulla or paraspinal ganglia. Sympathoadrenal progenitor cells express *anaplastic lymphoma kinase (ALK)*, which encodes a tyrosine kinase receptor that is the most frequently mutated gene in neuroblastoma. Activating mutations in the kinase domain are common in both sporadic and familial cases. The oncogenic role of ALK has been extensively studied, but little is known about its physiological role. Recent studies have implicated ALK in neural crest migration and sympathetic neurogenesis. However, very few downstream targets of ALK have been identified. Here, we describe pathological activation of ALK in the neural crest, which promotes proliferation and migration, while preventing differentiation, thus inducing the onset of neuroblastoma. Understanding the effects of ALK activity on neural crest cells will help find new targets for neuroblastoma treatment.

## 1. Introduction

Neuroblastoma is a childhood cancer: 40% of patients are younger than one year old at diagnosis [1,2]. Tumours are found along the peripheral nervous system and are composed of undifferentiated neuroblastic cells. These cells most likely originate from sympathoadrenal progenitor cells, which derive from neural crest cells. Although neuroblastoma only represents 6–10% of all childhood cancers, it is the most common extracranial solid tumour in young children, accounting for 12–15% of childhood cancer-related deaths [2]. Clinical prognosis in neuroblastoma is heterogenous, with outcomes varying from spontaneous remission to treatment-resistant progressive disease [1,2]. Overall survival in clinical low- to intermediate- and high-risk disease is 85–90% and <50%, respectively. High-risk disease represents approximately 40% of all diagnoses [1,2]. This depends not only on the age of the child (>15-month-old children display poorer prognosis), but also on the stage and the genetic makeup of the tumour [3].

Until now, no single genetic marker is accurately reflective of the complex clinical scenarios described above, and a variety of chromosomal alterations are used or are entering clinical consideration as potential risk-stratifying biomarkers. Structural chromosomal alterations are common, including chromosomal deletions of 1p and 11q arms, 17q gain, 14q loss, and triploidy [4]. The first neuroblastoma predisposition gene identified was paired-like homeobox 2B (*PHOX2B*), found in a familial case of neuroblastoma [5]. *PHOX2B* encodes a transcription factor essential for autonomic nervous system development, where it functions in sympathoadrenal specification, supporting the idea that neuroblastoma arises due to pathological development in this neural-crest-derived lineage [6]. Missense variants in *PHOX2B* are known to be drivers of other neural-crest-related diseases, such as congenital central hypoventilation syndrome and Hirschsprung disease [1]. However, only 1–2% of all neuroblastoma cases are familial [7,8], and, of these, only about 6–10% can be attributed to *PHOX2B* mutations [1].

A second gene, *MYCN,* is directly linked to poor prognosis in neuroblastoma patients [9,10]. Somatic amplification of the *MYCN* gene is found in 20% of all neuroblastoma patients, which results in higher MYCN protein levels [11]. In 1997, transgenic overexpression of *MYCN* in neural crest cells was shown to cause neuroblastoma in mice, helping to clarify the role of MYCN in the genesis of this disease [12].

Here, we focus on anaplastic lymphoma kinase (*ALK*), which is the most frequent single-gene alteration encountered in primary neuroblastoma. Heterogeneity at the *ALK* locus is found in almost all familial neuroblastoma cases [8,13,14,15] and is associated with poor prognosis in sporadic cases [16]. Subsequent tissue-specific *ALK*-overexpressing mouse models have been constructed that demonstrate synergy with *MYCN* in the ability to generate neuroblastoma [17,18] and have helped to clarify the oncogenic role of ALK, but, similarly, little is known about its physiological function. In this review, we will summarise the current understanding of ALK function and consider its potential role in neural crest development and in the onset of neuroblastoma tumorigenesis.

## 2. ALK Protein Structure and Interactions

**Figure 1:**
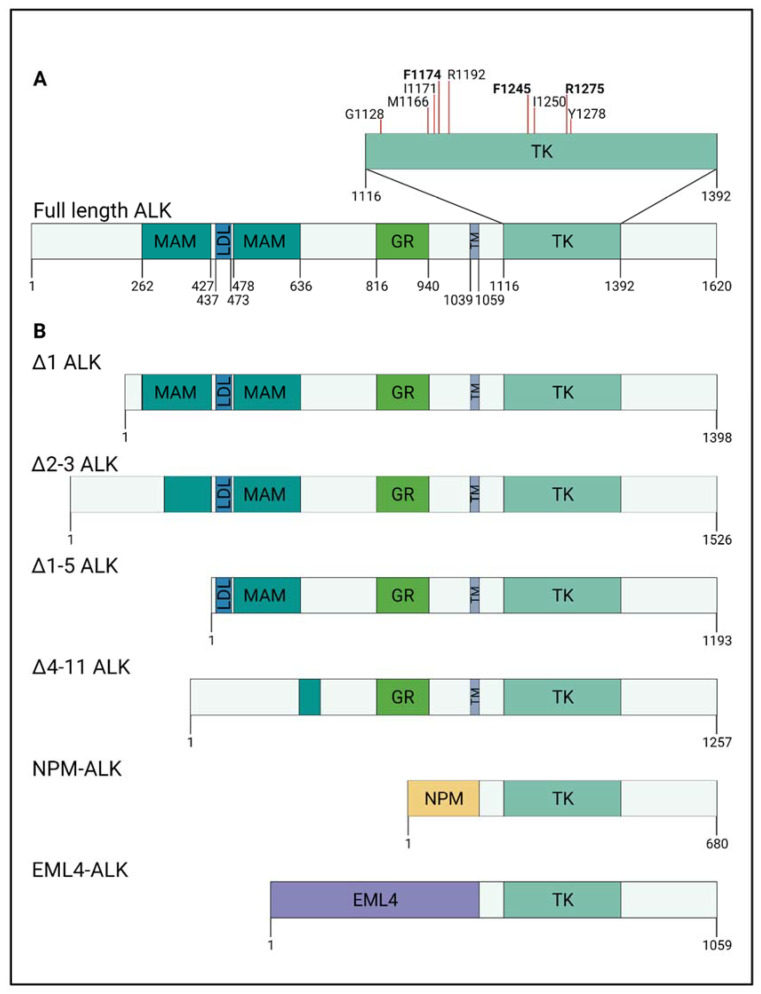
*ALK* mutations and alterations in neuroblastoma and other cancers. (**A**) Structure of ALK and neuroblastoma-associated mutations: the extracellular domain of ALK is composed of two meprin, A-5 protein, and receptor protein–tyrosine phosphatase mu (MAM) domains surrounding a low-density lipoprotein receptor class A (LDL) domain, a glycine-rich (GR) domain, transmembrane (TM) domain, and an intracellular tyrosine kinase (TK) domain. Neuroblastoma-associated mutations of ALK are found within the TK domain, and the majority of these lie in three distinct positions (bold font): R1275 (43%), F1174 (30%), and F1245 (12%). (**B**) Truncated ALK in neuroblastoma and fusion ALK proteins in other cancers. Four different truncated ALK proteins have been found in neuroblastoma. This is caused by translocation of *ALK*, leading to exclusion of *ALK* exons. *ALK*^Δ^^2–3^, *ALK*^Δ^^1–5^, and *ALK*^Δ^^4–11^ display altered or loss of MAM/LDL domains, which lead to constitutively active ALK. ALK fusion proteins NPM–ALK and EML4–ALK have been found in anaplastic large cell lymphoma (ALCL) and non-small cell lung cancer (NSCLC), respectively. The break point in *ALK* lies in introns flanking exon 16 and 17. This results in ALK fusion proteins displaying a complete loss of the extracellular domain and the TM domain.

ALK is a receptor tyrosine kinase (RTK) first identified in anaplastic large cell lymphoma patients, where *ALK* translocations led to an aberrant fusion protein with nucleophosmin (NPM) (discussed in more detail below, Morris et al., 1994). ALK, together with leukocyte tyrosine kinase (LTK), forms the LTK receptor subfamily [19,20]. Structurally, ALK comprises an extracellular glycine-rich domain, a transmembrane domain, and an intracellular tyrosine kinase domain (Figure 1).

In human ALK, the extracellular domain (ECD) is uniquely composed of two meprin, A-5 protein, and receptor protein–tyrosine phosphatase mu (MAM) domains, which surround a low-density lipoprotein receptor class A (LDL) domain (Figure 1). In contrast, the closely related human LTK has neither MAM nor LDL domains [21,22]. While these ALK domains have been identified, the complete structure and functions of the ECD still need clarification [20,23]. In other proteins, MAM domains are involved in cell–cell interactions through homophilic binding [24]. The LDL domain is likely to bind to ligands [25]. While full-length ALK protein is approximately 180 kDa, N-glycosylation of the extracellular domain increases the protein to a molecular weight of 220 kDa [20,26]. Altogether, the ECD is divided into presumed functions of ligand binding, interactions with potential coreceptors and regulatory proteins, and dimerisation [27].

The intracellular domain of ALK consists of the juxtamembrane region [28] and the tyrosine kinase domain [20]. The kinase domain has a small amino-terminal lobe and a large carboxy-terminal lobe and is highly conserved across species [29] (Figure 1). The kinase activity is dependent upon the catalytic loop, activation loop, αC-helix, and the glycine-rich region [29,30]. In the absence of a ligand, ALK has an autoinhibitory conformation, where the juxtamembrane domain clamps the αC-helix in a quiescent state with the activation loop, blocking substrate binding [31]. In addition, absence of a ligand induces the cleavage of ALK within the juxtamembrane domain by caspase-3. This cleavage releases an intracellular ALK fragment, shown to enhance apoptosis [28]. 

Upon ligand binding to the ECD, ALK undergoes dimerisation or oligomerisation, leading to activation of the tyrosine kinase [27,31] and transphosphorylation of one or more tyrosine residues in the juxtamembrane domain (Y1078, Y1092, Y1096, Y1131) [31]. Phosphorylation of the activation loop leads to a conformational change, allowing the active state of αC-helix [31]. In contrast to other receptor tyrosine kinases, ALK almost exclusively phosphorylates the first tyrosine of the Y-x-x-x-Y-Y motif [32]. Followed by ALK kinase activation, additional tyrosine residues in the C-terminus of ALK are phosphorylated, which function as docking sites for downstream targets [33].

Due to its high substrate specificity, not many ALK ligands have been found. In *Drosophila melanogaster*, jelly belly (Jeb), a secreted protein, has been identified as an Alk ligand. However, Jeb is unable to activate the mouse ALK orthologue, suggesting this interaction is not evolutionarily conserved [34]. In *C. elegans*, HEN-1 has been shown to bind and activate SCD-2, an ALK orthologue. This sensory pathway modulates transforming growth factor-β (TGF-β) signalling in response to environmental cues [35]. More recently, a screen for extracellular proteins identified two mammalian cytokines capable of binding LTK: family with sequence similarity 150 member A and member B (FAM150A and FAM150B). FAM150A binds LTK with high affinity and induces its phosphorylation [36]. However, FAM150A was shown to bind only weakly to ALK, while FAM150B binds to both ALK and LTK [37]. Moreover, in studies using zebrafish larvae, the three *Danio rerio* Fam homologues are all able to activate Ltk [38]; however, it is worth noting that, in zebrafish, Ltk is more similar to human ALK.

In primary neuroblastoma tumours, two neurotrophic factors, pleiotrophin (PTN) and midkine (MDK), are highly expressed and have been postulated to bind and activate ALK in vitro [39,40]. *PTN* seems to be expressed in samples from earlier stages and inversely correlates with *MYC* amplification, while *MDK* is similarly expressed across different stages [41]. However, there is conflicting evidence in the literature. Mathivet et al. showed that PTN failed to activate ALK in comparison to monoclonal antibody activation [42,43]. Moreover, Miple1 and Miple2, *Drosophila* homologues of the PTN/MDK family, are not required for embryonic development and failed to activate human ALK in vivo [44]. Nevertheless, PTN has been shown to increase ALK phosphorylation via the PTN/receptor protein tyrosine phosphatase β/ζ (RPTPβ/ζ) signalling pathway [45].

As an added complication, ALK can be considered a “dependence” receptor: rather than ligand binding acting as an on/off switch, the receptor may have ligand-independent and ligand-dependent functions [46]. In the case of ALK, it appears that ligand binding promotes signalling pathways driving survival, migration, and differentiation. In contrast, the absence of ligand can lead to cleavage of the receptors within the intracellular domain, with the generated intercellular fragment released into the cytosol and activating proapoptotic pathways [47]. This raises the possibility that the consequences of activating mutations in ALK are very much dependent on context, including availability of extracellular cues, such as ligands, and intracellular signal integration, and that the main consequence of ectopic activation is a failure to progress through the differentiation programme.

## 3. Developmental Roles of ALK

### 3.1. Expression Patterns of ALK

The role of ALK in embryogenesis is poorly understood, but its expression pattern during embryonic development across different animal models points to a function in the developing nervous system. In *Drosophila*, the *Alk* orthologue is expressed in the developing nervous system, as well as in the visceral mesoderm [48]. In chick embryos, *ALK* is expressed in the peripheral nervous system, in the sympathetic and dorsal root ganglia of the spinal cord, specifically in motor neurons from stage HH19 to HH39 [49,50]. However, in chicken, LTK is more structurally similar to human ALK and could be functionally more similar to human ALK than human LTK. Chicken *LTK* is expressed in the neural plate border and in migrating cranial and trunk neural crest cells [51]. 

In mice, ALK protein and mRNA are seen in the brain when assessed in 1-day postnatal (P1) to 2-week-old (P14) animals. In older mice, expression levels appear low. Transcripts were also observed in the central and peripheral nervous system in embryonic day 15 and 19 mice (E15 and E19) [52]. Earlier in foetal development (E10.5-E16.5), *Alk* expression was detected in neuronal tissues, in the brain and spinal cord, as well as in gastrointestinal tissues, the lining of the gut, and in sensory organs, such as the tongue, skin, and the nasal epithelium, and testis and ovaries [53]. Finally, Gonzalez Malagon et al., showed ALK protein and mRNA expression in the neural plate border of E8 to E9.5 mice and in migratory neural crest cells in explants of E8.5 embryos [54]. These stages correspond to neural crest delamination and migration, and may be most relevant to neuroblastoma.

More recently, our group described the expression pattern of *alk* during embryonic development of *Xenopus*. We found that *alk* is expressed in neural crest domains as early as stage 13 (prior to neural tube closure), in the neural plate and in migrating neural crest streams in the head, until later tadpole stages. Expression was also seen in other ectodermal derivatives, such as ear vesicles, optic cup, in the lining of the pharynx, and in head mesenchyme [55,56]. All in all, *alk* is expressed in the nervous system, particularly in neural crest cells. Nevertheless, how Alk functions in these cells remains unclear.

### 3.2. Developmental Roles of ALK

While endogenous functions of ALK have mainly been examined in disease contexts, substantial studies in *Drosophila* have linked Alk to gut development [57,58], eye development [23], protection of neuroblasts during nutrient deprivation, and to a role in alcohol resistance [59]. Both Alk and the Jeb ligand play roles in muscle founder cell specification by inducing dumbfounded (*duf*) expression through extracellular signal-regulated kinase (Erk) and Ras/mitogen-activated protein kinase (Ras/Mapk) activation [57,60]. In *C.* elegans, SCD-2 and its ligand HEN-1 are implicated in associated learning and sensory integration [61,62,63]. 

Although expression in humans and mice suggests that ALK is involved in nervous system development, deletion of *Alk* in mice does not lead to obvious anatomical phenotypes and, therefore, the requirements for ALK in mammals are still unclear [23]. However, *ALK* depletion does lead to changes in neurogenesis and neuronal proliferation in several animal models. In vitro experiments with immature sympathetic neurons from chickens displayed increased proliferation dependent on ALK activity [64]. In vivo, *ALK* knockout leads to a reduction in sympathetic ganglia size and reduction in proliferating immature sympathetic neurons [64]. As noted, both chicken and zebrafish Ltk is structurally more similar to human ALK, and zebrafish Ltk plays a role in neural crest cell differentiation into eye iridophores [38]. Mutations in *ltk* or loss-of-function of either FAM homologue, *alk* or *ltk*, all result in loss of iridophore patterning, while overexpression of the ligands drives ectopic iridophore formation in vivo [38,65,66].

## 4. ALK in Neuroblastoma

### 4.1. Fusion Proteins NPM–ALK and EML4–ALK Are Common in Cancer

While the physiological role of ALK is not well understood, genomic variations in *ALK* have been known for over 20 years. Nearly 30 different ALK fusion proteins have been described, in which NPM–ALK and EML4–ALK are the most studied [67]. Morris and colleagues first discovered ALK in 1994 as a fusion protein with nucleophosmin (NPM) in anaplastic large cell lymphoma (ALCL) (Figure 1), which is a non-Hodgkin lymphoma [19]. EML4–ALK was described in 2007 in non-small cell lung cancer (NSCLC), where ALK is fused to echinoderm microtubule-associated protein-like 4 (EML4) [68,69]. ALK fusion proteins have also been described in diffuse large B-cell lymphoma, inflammatory myofibroblastic tumour, oesophageal squamous cell carcinoma, serous ovarian carcinoma, and breast and colon cancer [70].

ALK fusion proteins are able to oligomerise, which is mediated by ALK protein partners [67]. They can gain additional functional and interaction partners mediated by its fusion partner [71]. As the break point of *ALK* in every fusion is in the intron flanking exon 16 and 17, this leads to ALK fusion proteins containing the active intracellular region of the ALK kinase domain. In NPM–ALK, this leads to a ligand-independent, constitutive tyrosine kinase activity [70].

### 4.2. ALK Alterations in Neuroblastoma

To date, no fusion proteins have been reported in neuroblastoma. However, several groups describe *ALK* rearrangements and amplification (Table 1). *ALK* rearrangements are found in 23% of investigated neuroblastoma cell lines and a subset of neuroblastoma tumours [72]. Miyake et.al. (2002) described *ALK* amplification in the NB-39-nu neuroblastoma cell line, which leads to constitutive activation of ALK [73]. NB-1 neuroblastoma cells exhibit *ALK* amplification, in addition to truncated *ALK*^Δ2–3^ caused by deletion of exon 2 and 3 [74]. Other truncations seen in neuroblastoma cell lines include *ALK*^Δ4–11^, which lacks a partial MAM domain, the complete second MAM domain, and the LDL domain [72], and *ALK*^Δ1–5^, which lacks the first MAM domain and part of the LDL domain. All three *ALK* deletion variants act in a ligand-independent manner and are constitutively active, with *ALK*^Δ1–5^ also showing downstream phosphorylation of ERK increases upon stimulation [75].

Gain-of-function mutations in *ALK* are the predominant driver of familial neuroblastoma (Table 2). The majority of these are found in three distinct positions located in the kinase domain: R1275 (43%), F1174 (30%), and F1245 (12%) [16,76,77]. R1275 mutations are present in familial and sporadic cases, whereas F1174 and F1245 are mostly found in sporadic cases [16,76,77]. They are associated with an aggressive tumour phenotype, and poor prognosis and survival [76,78]. Mutations in R1275 and F1174 lead to a ligand-independent activation of ALK, with increased kinase activity [13,14,15,27,79,80,81]. Other mutations are found in I1170 (N or S), I1171N, and 15 further positions [8,76,82,83,84,85,86] (Table 1).

### 4.3. ALK and MYCN in Neuroblastoma

A key question in the field is whether *ALK* mutations themselves can initiate tumour formation or whether other genomic alterations are required. *ALK^F1174L^* expression in transgenic mice led to some tumour formation resembling human neuroblastoma [18]. However, this was challenged in 2014, when Cazes et al. showed that *ALK^F1174L^* and *ALK^R1275Q^* variants indeed lead to abnormal proliferation of sympathetic ganglia, but were not sufficient to initiate neuroblastoma formation. This suggests a requirement for additional factors, such as *MYCN* amplification, to trigger tumorigenesis [89,90]. Increased MYCN activity is likely to enhance neuroblast proliferation and survival [91]. Following this, ALK activation may potentiate the oncogenic capacity of MYCN [17,90,92,93,94]. These two factors are the most common predisposition markers of neuroblastoma and, together, are associated with poor prognosis [77,95]. 

MYCN is a transcription factor expressed early during neural crest induction [96] and is thought to promote cell expansion during normal murine sympathoadrenal development [97]. However, *MYCN* expression decreases during sympathetic neuron differentiation [97] and no expression is detected in adult neural tissue [11]. Intriguingly, neural crest cells with forced *MYCN* expression do not lose their ability to migrate but, rather, are unable to differentiate normally [98].

*ALK* and *MYCN* are located in close proximity on chromosome 2, at 2p23 and 2p24.1, respectively [99]. This appears to be a region of genomic instability. Gains of the 2p chromosome are associated with *MYCN* and *ALK* amplification and, again, correlate with poor prognosis. Amplification of *ALK* has been found almost exclusively concomitantly with *MYCN* amplifications [76,99]. In 10.9% of neuroblastoma tumours with *MYCN* amplification, additional *ALK* mutations were found [76]. In total, 41% of these tumours exhibit *ALK^F1174L^* mutations compared with other *ALK* mutations [76,77]. ALK activation also leads to *MYCN* transcription [100], whereas MYCN reciprocally regulates *ALK* expression [92].

Interestingly, *FAM150B* is also located in the chromosome 2p arm along with *MYCN* and *ALK*. Thus, it is possible that, in some cases, an amplification of *FAM150B* may play a synergistic role in tumorigenesis, resulting in enhanced ligand-dependent activation of wild-type ALK [101]. In fact, overexpression of *FAM150B* in the *Th-Mycn* neuroblastoma mouse model (which has no ALK-activating mutations) induced tumours that are sensitive to tyrosine kinase inhibitor (TKI) treatment, while ALK mutations confer resistance [102].

## 5. Downstream Targets of ALK

**Figure 2:**
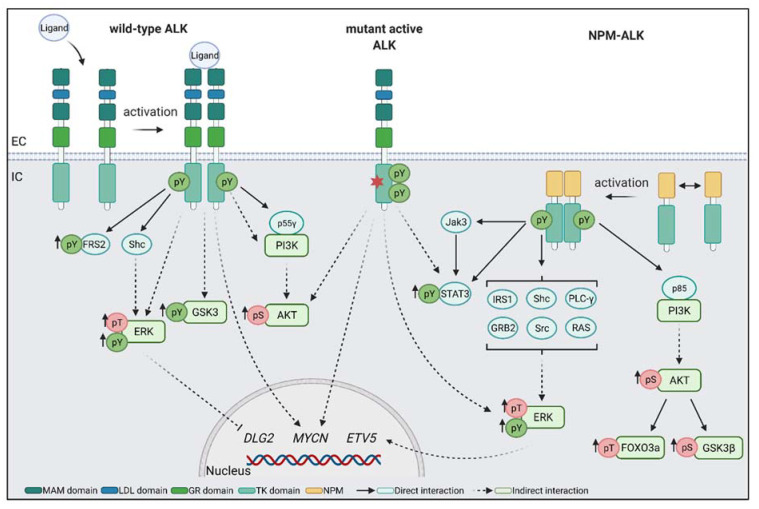
Downstream signalling of wild-type ALK, mutant active ALK, and NPM–ALK. Wild-type ALK activation is dependent on ligand binding, leading to activation and transphosphorylation of tyrosine residues in the tyrosine kinase (TK) domain. Phosphorylated tyrosines (pY) can function as docking sites for downstream targets, e.g., Shc and p55γ. p55γ is a regulatory subunit of PI3K and its activation leads to increased phosphorylation of AKT. Binding of Shc to pY-ALK leads to activation of the MAPK/ERK pathway and increased phosphorylation of ERK1/2. Both pathways are used as readouts of ALK activity. Mutations in the ALK tyrosine kinase domain can lead to constitutively active ALK independent of ligand binding. In neuroblastoma cells, mutated ALK may lead to increased STAT3 phosphorylation. In addition, in neuroblastoma cell lines with elevated active ALK, increased levels of phospho-tyrosine GSK3 have been shown. NPM–ALK fusions are found in anaplastic large cell lymphoma. NPM–ALK activity is dependent on the dimerisation of the fusion protein NPM. Similar to wild-type ALK, dimerisation leads to auto-phosphorylation of the tyrosine kinase domain. IRS1, Shc, PLC-γ, GBR2, and RAS have been shown to directly bind to NPM–ALK, which leads to activation of the MAPK/ERK pathway. In addition, NPM–ALK has been shown to directly bind and phosphorylate STAT3. In contrast to wild-type ALK, NPM–ALK has been shown to favourably bind to p85, a regulatory subunit of PI3K, and thereby activate the PI3K/AKT pathway.

Downstream targets of ALK have mainly been studied in pathological disorders, such as ALCL, and in the context of ALK fusion proteins, such as NPM–ALK. However, these fusion proteins have altered intracellular localisation, having lost the extracellular and transmembrane domains [71]. In these pathological conditions, ALK reportedly activates MAPK/ERK and phosphoinositide 3-kinase/protein kinase B (PI3K/AKT) pathways (Figure 2). Additionally, phosphorylation of ERK1/2 is widely used to determine ALK activation in in vitro assays [17,87,103,104].

The fusion protein NPM–ALK can directly interact with a variety of proteins. The regulatory subunit p85 of PI3K binds directly to NPM–ALK, resulting in the activation of the PI3K/AKT pathway [105]. The PI3K/AKT pathway is known to be involved in cell proliferation and survival. Through activation of AKT and mTOR complex 1 (mTORC1), PI3K promotes cell growth in neural crest cells [106]. In addition, PI3K activation leads to increased FOXO3a phosphorylation, which affects cell survival and proliferation [107]. In neuroblastoma, FOXO3a is proposed to act as a tumour suppressor due to its reduced expression in aggressive tumours. Inhibition of AKT leads to reactivation of FOXO3a and induces apoptosis in neuroblastoma cell lines [108]. Activation of the PI3K/AKT pathway mediated by NPM-ALK has also been shown to phosphorylate serine 9 of glycogen synthase kinase 3 isoform β (GSK3β), which inhibits GSK3 kinase activity [109]. In neural crest development, these inhibitory phosphorylations of GSK3 lead to Wnt signal activation and neural crest induction [110]. Moreover, our group showed that neuroblastoma cell lines with elevated (active) tyrosine-phosphorylated ALK present increased levels of (active) tyrosine-phosphorylated GSK3α and GSK3β [54], raising the possibility that GSK3 may be a direct phosphorylation target of ALK.

In addition, NPM–ALK has been shown to directly bind to insulin receptor substrate 1 (IRS1) [111], SHC [103], phospholipase C γ (PLC-γ) [112], growth factor receptor-bound protein 2 (GRB2) [113], and proto-oncogene tyrosine–protein kinase src (SRC). These proteins are involved in MAPK/ERK activation. In neural crest induction, the MAPK/ERK pathway is activated by the fibroblast growth factor receptor (FGFR) [106]. Interestingly, mutations in MAPK/ERK activation pathways are frequently observed in neuroblastoma relapse samples [114]. Finally, NPM–ALK has been shown to mediate the phosphorylation of signal transducer and activator of transcription 3 (STAT3). STAT3 is a transcription factor, which activates and regulates genes involved in proliferation, apoptosis, and differentiation [115,116]. In neuroblastoma cells, STAT3 has been found to directly bind to full-length ALK, which leads to STAT3 phosphorylation. Interestingly, inhibition of STAT3 leads to decreased growth and viability of neuroblastoma cell lines, in addition to decreased levels of MYCN in neuroblastoma cell lines carrying ALK mutations [117].

A few studies show downstream targets of wild-type ALK. In developing mouse brains, endogenous ALK has been found to regulate migration through p55γ, another regulatory subunit of PI3K [118]. In vitro experiments with overexpression of ALK in PC12 cells show increased phosphorylation of ERK1/2 upon ALK activation [43]. In addition, wild-type ALK activation in HEK293 cells leads to the specific activation of STAT3 over STAT1 and STAT5 [43]. 

Although these studies tell us clearly which intracellular signals can be activated by the ALK kinase, it is hard to pinpoint the precise tissue- and cell-type requirements for ALK signalling. As noted here, many studies focus on ALK fusion proteins, or on ALK gain-of-function mutations, which confer ligand independence and promote contextually inappropriate activation of intracellular effectors. This can make it difficult to determine which signalling pathways are crucial for normal ALK function. Furthermore, the effects may be twofold: first, a loss of the ligand-independent signals, e.g., those that may drive apoptosis, coupled with the gain of prosurvival, promigratory signals. 

Moreover, ALK binding partners or downstream pathways described above are also known to be activated by other RTKs important in neural crest development [6,119]. To date, there is little known about ALK specific signalling to postulate its precise role in this signalling network. Nevertheless, gain-of-function mutations in ALK can lead to a disease outcome, emphasizing that ALK has a specific role in the neural crest development signalling network. For future research, it will be important to understand ALK specific signalling and how we can distinguish it from other RTK activated pathways. This knowledge will help us to develop better strategies for ALK-positive neuroblastoma treatment.

## 6. ALK in Neural Crest and Neuroblastoma

Despite many decades of work, the cell of origin of neuroblastoma has not been clearly defined; however, it is generally accepted that neuroblastoma arises from trunk neural crest cells contributing to the sympathoadrenal lineage. The sympathoadrenal lineage is mainly comprised of sympathetic neurons and chromaffin cells of the adrenal medulla [120,121]. Evidence from early mouse and chicken models have shown that both of these cell types share a common sympathoadrenal progenitor (SAP), which is fate restricted [122,123,124,125]. Differentiation from SAPs to sympathetic neurons and its implications in neuroblastoma have been extensively studied [121,126]. Recently, Furlan and colleagues have demonstrated that a part of chromaffin cells originate from a Schwann cell precursor (SCP) via a transitory “bridge cell” stage [127,128]. However, questions remain regarding chromaffin cell differentiation from SAP/SCPs and whether this cell type can develop into neuroblastoma.

**Figure 3:**
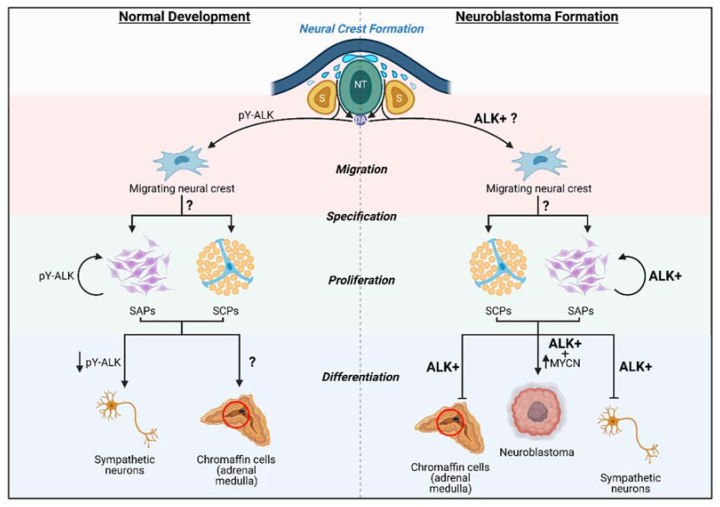
ALK in neural crest development and neuroblastoma formation. Normal development: neural crest progenitors arise from the dorsal neural tube. Cells undergo epithelial-to-mesenchymal transition, delaminate from the neural tube, and migrate, either laterally, adjacent to the somites (S), or towards the dorsal aorta (DA). The DA population of trunk neural crest cells start to specify into sympathoadrenal progenitor (SAP) cells or Schwann cell progenitors (SCP). These cells will eventually give rise to sympathetic neurons or chromaffin cells, respectively. Activated ALK (pY-ALK) can be found in migrating neural crest cells and is shown to be important in proliferation of SAP cells, but less so as they differentiate into sympathetic neurons. Neuroblastoma formation: *ALK* alterations (mutations, amplification, or translocation) can lead to constitutively active ALK (ALK+). During migration of neural crest cells, ALK+ is thought to increase their migratory behaviour. In addition, ALK^F1174L/R1275Q^ have been shown to prolong SAP proliferation, which leads to the blockage of the differentiation into sympathetic neurons. The effect of ALK activation in SCPs and its involvement in neuroblastoma remains unclear. Recently, it has been proposed that ALK alterations potentiate MYCN oncogenic capacity, resulting in neuroblastoma formation [17,90,92,93,94,127,128]. NT = neural tube, DA = dorsal aorta, S = somite, pY-ALK = active ALK, SAP = sympathoadrenal progenitors, SCP = Schwann cell precursors, ALK+ = constitutively active ALK.

Regardless, neuroblastoma prognosis and aggressiveness are closely linked to the tumour differentiation state. We can postulate that an arrest in neural crest cell development results in undifferentiated stem-like cells, which lead to highly metastatic neuroblastoma (Figure 3). This malignant transformation could be promoting proliferation and migration, while blocking final differentiation of neural crest cells [54]. This is consistent with data from *ALK^F1174L^* knock-in mice, which display prolonged sympathetic neurogenesis with increased levels of the proliferation marker Ki67 [89]. In contrast, ALK knockdown leads to the reduction in proliferating immature sympathetic neurons, in addition to decreased sympathetic ganglia size [64].

Evidence linking pathological activation of ALK with neuroblastoma cell migration has been limited [92]; nevertheless, there have been a sprinkling of papers. Overexpression of *ALK* mutants or fusions results in increased migration and invasiveness in neuroblastoma cells via upregulation of the MAPK pathway target *ETV5* [129]. Indeed, using chicken embryo grafting experiments, Delloye-Bourgeois and colleagues were able to show that neuroblastoma cells carrying *ALK* gain-of-function mutations could follow the neural crest migratory paths, but did continue to proliferate and form tumour-like masses [130]. 

In addition, mouse models support a role for ALK early in neural crest development. In mice, as in humans, migratory neural crest cells express the transcription factor *Sox10* (*Sox10^+^/Phox2b^−^*). As these cells mature to the sympathetic neuroblast lineage, *Sox10* is downregulated and *Phox2b* is activated (*Sox10^−^/Phox2b^+^*). Normally, expansion of these *Phox2b+* cells occurs dramatically between E10.5 (43.1%) and E11.5 (84.4%) [78]. In mice carrying a *Sox10* promoter-driven Cre-recombinase and a conditional *ALK^F1174L^* variant (*Sox10::CreERT2/+*; *LSL-ALK^F1174L^)*, neural crest migration remained unaltered. However, the transition from *Sox10^+^* migratory cells to *Phox2b^+^* neuroblasts was not observed, with roughly equivalent numbers of *Sox10^+^*-only cells (32.5%), *Sox10^+^/Phox2b^+^* double-positive cells (29.9%), and *Phox2b^+^*-only cells (37.6%) equally represented [78]. Interestingly, *ALK^F1174L^* expression before lineage specification led to 100% lethal in E12.5 mice and blockage of noradrenergic differentiation [78], pointing to a specific role of ALK after sympathoadrenal lineage specification.

Together this might suggest that ALK activation is involved in NCC migration and proliferation, however, not required for differentiation. In fact, through mutation, ALK activation uncoupled from ligand binding may result in a blockage of differentiation, leading to the hypothesis that ectopic ALK signalling acts very early in the neural crest differentiation programme when initiating neuroblastoma. This was given emphasis when Siaw and colleagues proposed that ALK activation in neuroblastoma can inhibit *DLG2* transcription via ERK1/2 and specificity protein 1 (SP1) signalling, thus impairing DLG2-induced differentiation. DLG2 expression is shown to inhibit tumour growth in vivo and drive neuroblastoma cell differentiation [131]. Previous studies have described DLG2 expression in the “bridge cell” signature between Schwann cell precursors (SCP) and Schwann cells, and late bridge cell signatures in neuroblastoma tumours have been linked to better prognosis [127].

Histological assessment of the tumours has allowed clinicians to establish a classification of risk status and prognosis of disease, with undifferentiated tumours being categorised as high-risk, associated with worse prognosis and survival [132,133,134]. *ALK* genetic abnormalities (mutation or amplification) have been associated with high-risk neuroblastoma, with tumours often composed of undifferentiated neuroblasts instead of differentiated neuronal cells [95,135].

Despite intense research, our knowledge about ALK is limited. Through mRNA expression patterns in a variety of animal models [48,49,52,55], we know that ALK is expressed in neural crest cells during early development. Forced overexpression or deletion of ALK in neural crest cells leads to changes in neural crest migration [129], differentiation [131], and proliferation [89]. Nevertheless, we are still not able to postulate the exact role of ALK in the neural crest.

In addition, we are lacking in human models that are not disease based. Currently, we are using a variety of human disease and animal models to establish the role of ALK in development and neuroblastoma initiation. These might not reflect essential endogenous roles of ALK. A better understanding of the normal regulation and function of ALK during human neural crest development is crucial for understanding disease progression. Further, the design of in vitro and in vivo models with inducible ALK expression during different developmental stages is essential. These models would help us to elucidate ALK’s role in neural crest development, neuroblastoma initiation, and treatment development.

## 7. Treatment of ALK-Positive Neuroblastoma

So far, a variety of neuroblastoma treatments have been established, which vary depending on the neuroblastoma risk groups (reviewed in Matthay et al., 2016). These treatments include surgery, chemotherapy, and radiotherapy, but also differentiation therapy and immunotherapy. Although ALK has been associated with high-risk neuroblastoma and a variety of ALK inhibitors are available, none of these are currently approved to treat ALK-positive neuroblastoma. In fact, *ALK*-mutated tumours can be highly susceptible to resistance to first-generation tyrosine kinase inhibitor therapies, such as crizotinib, or second-generation therapies, such as brigatinib or ceritinib [136]. However, ALK activity can also be targeted indirectly, using inhibitors against downstream effectors, e.g., PI3K/AKT, MAPK/ERK, or MYCN. *ALK* alterations have been frequently observed in relapse cases of neuroblastoma [137]. Further, mutations in the downstream target MAPK/ERK pathway are frequently found in relapse neuroblastoma tumours [114]. More promising are differentiation therapies for neuroblastoma [138], where retinoic acid (RA) treatment can overcome the differentiation block in some patients. Retinoic acid is known to upregulate (rearranged during transfection) RET receptor [139], which is a proposed phosphorylation target of ALK in sympathetic neurons [89,140,141]. Furthermore, multiple studies are investigating combinatorial treatments for ALK and its downstream targets [142].

Neuroblastomas with ALK alterations and MYCN amplifications are mostly classified as high-risk tumours with poor prognosis [77,95]. Expression levels of ALK and MYCN are tightly connected. Via the PI3K/AKT pathway, ALK activates MYCN expression, whereas ALK is a transcriptional factor of MYCN [92,100]. This unique relation can be used to generate combinational treatment approaches. Currently, there are no inhibitors for MYCN available; however, indirect inhibitors for its cofactors and downstream targets have shown some clinical benefits [143]. For example, GSK3β has been found to directly phosphorylate and destabilize MYCN [144], and, as noted, ALK is suggested to phosphorylate GSK3 (Gonzalez Malagon, et al., 2018). Most promising GSK3 inhibitors being developed are still in preclinical/clinical trials [145]. Inhibiting signalling pathways, which would phosphorylate and, thereby, inhibit GSK3β, would result in increased MYCN degradation. However, it is still essential to understand the developmental biology and the signalling events in the neural crest that depend upon ALK to improve neuroblastoma treatments.

A variety of studies concentrate on defining the ALK interactome in neuroblastoma cells and have identified new major interacting partners [104,117,146]. Using mass spectrometry, Sattu et al. and Emdal et al. investigated differences in the phosphoproteome of ALK, dependent on genetic alterations or ALK inhibition in neuroblastoma cell lines [104,117]. A membrane-specific two-hybrid approach identified proteins that bound to ALK in a phosphorylation-dependent ALK manner [146]. Using these approaches, they were able to define an ALK interactome, including novel targets, such as NCK2, which were then validated in neuroblastoma cell lines carrying activated ALK. NCK2 is an SH2-domain-containing protein that binds to actin effectors, suggesting a link between ALK and regulation of the cytoskeleton during cell migration. Another elegant study combined cellular fractionation to determine tyrosine kinase and phosphatase interactions in neuroblastoma cell lines. They were able to determine that localisation of FYN and LYN kinases changed in response to ALK and KIT stimulation (Palacios-Moreno et al., 2015). As both ALK and KIT are associated with aggressive neuroblastoma and naïve neural crest, Palacios-Moreno and colleagues propose that these sustained phospho-tyrosine signatures are indicative of a failure to differentiate (Palacios-Moreno et al., 2015). It will, of course, be important to test the relevance of similar ALK interactions during normal neural crest development and migration.

## 8. Summary and Long-Term Prospects

Childhood cancers are often thought of as developmental cancers. Compared to adult cancers, childhood cancers often have fewer associated genetic mutations. Presumably, this is because residual embryonic cells carry the hallmarks of cancer “stem cells”: namely, they are proliferative, migratory, resistant to apoptosis, and have self-renewal capacity. Neuroblastoma is particularly interesting because of the heterogeneity of disease outcomes, from metastasis to spontaneous regression, and because there are a few genes associated with the most severe cases.

Although, *ALK* is a known predisposition gene for neuroblastoma development, we are still far from understanding its role in tumour initiation. Most of the current research has been in a disease context or is based on overexpression, deletion, or loss-of-function assays. In addition, our understanding of the endogenous role of ALK in neural crest development is limited and inhibits us from fully understanding its involvement in neuroblastoma formation. For future research, we need to determine the effects of “normal”, gain-of-function mutation and amplified ALK expression/activation during neural crest development. Currently, our understanding is that endogenous *ALK* and function should be restricted to specific developmental contexts. However, due to the overactivation of mutated ALK and its downstream signals, the proliferative and migratory phases are prolonged and expression of differentiation genes are blocked. This can lead to undifferentiated tumour-like cells. This is exacerbated in combination with other gene alterations, such as *MYCN* amplifications, which then can lead to neuroblastoma formation. In comparison, *ALK* amplification leading to unrestricted expression of *ALK* during undefined developmental stages results in the severe disruption of specification and consequent tumour formation, or death during development, as seen in *Sox10::CreERT2/+*; *LSL-ALK^F1174L^* mice [78]. A more precise definition of the role of ALK during neural crest development will be crucial to our understanding of the initiation of ALK-positive neuroblastoma and will help us to develop new treatment strategies to correctly treat neuroblastoma and potentially decrease the risk of relapse.

## Figures and Tables

**Table 1:** *ALK* alterations described in neuroblastoma.

	Alteration	Affected Domain	Note	Reference
Amplification	2p23	Full-length ALK	Ligand-dependent	[14]
Translocation/amplification	Δ1	Extracellular N-terminal	Translocation to 11q14	[75]
	Δ2–3	Extracellular N-terminal	Enhanced kinase activity	[74]
	Δ1–5	Extracellular N-terminal	Translocation to 4q35 or 2p24	[75]
	Δ4–11	MAM and LDL domain loss	Ligand-independent kinase activity	[72,75]
	3–4 exon	Extracellular N-terminal	Translocation to 2p16–2p14 region	[75]

**Table 2:** *ALK* point mutations described in neuroblastoma.

Type	Mutation Site		Domain	Note	Reference
unknown	p.(K1062M)	AAG > ATG	Kinase domain (Juxtamembrane)	Tumorigenesis in mice	[14]
Germline	p.(T1087I)	ACC > ATC	Kinase domain (Juxtamembrane)	unknown	[14]
Germline	p.(G1128A)	GGG > GCG	Kinase domain (P-loop, glycine loop)	Ligand-independent kinase activity	[8,82,83]
Somatic	p.(M1166R)	ATG > AGG	Kinase domain (αC helix)	Ligand-independent kinase activity	[8,83]
Somatic	p.(I1171N)	ATC > ACC	Kinase domain (αC helix)	Ligand-independent kinase activity	[8,83]
Somatic	p.(F1174I)	TCC > ATC	Kinase domain (αC helix)	Ligand-independent kinase activity	[8,82]
Somatic	p.(F1174L)	TTC > TTA TTC > TTG TTC > CTC	Kinase domain (αC helix)	Ligand-independent kinase activity	[4,8,13,82]
Somatic	p.(F1174C)	TTC > TGC	Kinase domain (αC helix)	Ligand-independent kinase activity	[4,14,79]
unknown	p.(F1174S)	TTC > TCC	Kinase domain (αC helix)	Ligand-independent kinase activity	[80,86,87]
Somatic	p.(F1174V)	TTC > GTC	Kinase domain (αC helix)	Ligand-independent kinase activity	[4,14,81]
Germline	p.(R1192P)	CGG > CCG	Kinase domain (β4 strand)	Ligand-independent kinase activity	[4,8,83]
Somatic	p.(F1245C)	TTC > TGC	Kinase domain (catalytic loop)	Ligand-independent kinase activity	[8,13,83]
Somatic	p.(F1245L)	TTC > TTG	Kinase domain (catalytic loop)	Ligand-independent kinase activity	[14,76,84,88]
Somatic	p.(F1245V)	TTC > GTC	Kinase domain (catalytic loop)	Ligand-independent kinase activity	[8,13,76]
Somatic	p.(I1250T)	ATT > ACT	Kinase domain (catalytic loop)	Kinase dead mutation	[8,86]
Somatic/Germline	p.(R1275Q)	CGA > CAA	Kinase domain (activation loop)	Ligand-independent kinase activity	[4,8,13,14]
Somatic	p.(Y1278S)	TAC > TCC	Kinase domain (activation loop)	Ligand-independent kinase activity	[4,83,85]
